# The Advance of Gastroenterology
*Based on an address to the Bristol Medico-Chirurgical Society, 11th May, 1966


**Published:** 1966-10

**Authors:** J. M. Naish

**Affiliations:** Consultant Physician, Frenchay Hospital, Bristol


### THE ADVANCE OF GASTROENTEROLOGY*

J. M. NAISH, M.D., F.R.C.P.

*Consultant Physician, Frenchay Hospital, Bristol*

Gastroenterology may be defined as the body of knowledge relating to the functions of ingestion, digestion, absorption and elimination of food. A clinical gastroenterologist in 1920 might have been defined as a man who not only looked at the X-ray reports, but also at the films ! Today many distinguished gastroenterologists occupy chairs of medicine, of surgery, of therapeutics, of pathology and of physiology. Personalities are important in medicine, because of the impetus which they can give to the acquisition and dissemination of knowledge. Advances in the sphere of gastroenterology have been rapid in the post-war years, and the inspiration and enthusiasm of certain vigorous personalities in Britain has caused an exhilarating build-up of productive research. For this reason the story of the development of gastroenterology as a special branch of research and practice is worth telling.

In Bristol, 1966 is a happy year in which to make this report because W. M. Capper, Consultant Surgeon at the United Bristol Hospitals and Southmead Hospital, is the current President of the British Society of Gastroenterology which met for the first time ever in Bristol in November 1965. Not only this, but Alan Read, Reader in Medicine at Bristol University, has been elected the Secretary of the whole organisation. Thus Bristol is now playing a key role in the affairs of British gastroenterology.

### THE HISTORY OF GASTROENTEROLOGY AS A SPECIALTY

Our ancestors were rather crude in their approach to the gastro-intestinal tract. They could, of course, only get at it through the top and bottom end, a limitation of nature which still influences modern machinery such as the oesophagoscope, the gastroscope and the sigmoidoscope, but ingenious and resourceful gastroenterologists today have no hesitation in puncturing the skin in order to get at the gastro-intestinal tract.

It was in 1838 that gastricphysiology began to go ahead under the stimulus of Beaumont's accurate and careful studies of that famous gastrotomist, Alexis St. Martin. Beaumont observed the basic motility patterns of the stomach, and showed that the secretion of hydrochloric acid, previously shown to be a component of gastric juice by Prout in 1824 was, though under nervous control, a continuous process, mainly motivated by the presence of food in the stomach. Heidenheim developed a technique for making separate gastric pouches in dogs so that gastric secretions could be studied. The work of Pavlov coming soon afterwards stressed the importance of psychic factors in stimulating gastric secretion. The role of the vagus nerve as the transmitter of psychic stimulation to the gastric factory thenceforward became increasingly clear. But this was gastric physiology in animals, and it was nearly 1900 before an attempt was made to translate the knowledge gained into the sphere of human experiment and therapeutics. The Guy's Hospital partnership of Sir Arthur Hurst and John Ryle put the fractional test meal on the map, and though its clinical usefulness may in retrospect be doubted, at least they had moved on to the phase when knowledge was sought from measurements and experiments on human volunteers and willing patients. The demonstration that pernicious anæmia only occurred in the presence of complete achlorhydria, and that duodenal ulcer most often occurred in those with high concentrations of gastric acid were two of the basic and fundamental truths which came from this type of work.

At this time the development of X-rays and barium techniques greatly added to the possibilities of accurate clinical diagnosis. Meanwhile on the other side of the Atlantic where the idea of specialism in medicine has always been more readily welcomed than in our tight little island, the concept of a gastroenterologist as a species of human who made himself a master of such primitive techniques as were then available—barium flurosocopy of stomach and colon, gastric secretory analysis, endoscopy of rectum and

*Based on an address to the Bristol Medico-Chirurgical Society, 11th May, 1966

œsophagus, bacteriological and parasitological study of the stools, was rapidly gaining ground. Another reason why this happened so early in America is that tropical and parasitic diseases, so many of which affect the alimentary tract, are there more prevalent. Alcoholic cirrhosis also was ever commoner in the States — even in the days of prohibition. Thus in 1895 the American Gastroenterological Association was founded and so began that fruitful association of physiologists, pathologists, physicians, and surgeons in a few hospital centres. Towards these centres came patients from all over the continent, hopeful that their obscure conditions would be more accurately diagnosed and effectively treated by such experts. Their hopes were often disappointed, and it is probable that the standard of treatment of common gastroenterological conditions by general physicians and general surgeons in British hospitals was every bit as good, but at least these American centres had the chance to build up a tremendous library of clinical material, to found laboratories and research units dedicated to solving some of the thousands of unanswered questions, and to add to knowledge about the gastro-intestinal tract.

During the inter-war years one of the most important landmarks in the advance of gastroenterology was the working out that the causes of pernicious anæmia lay in the stomach, and the development of rational and effective treatment for this disease.

In 1932 Crohn described twelve cases of the disease which now bears his name, but let us not forget that Jackman of Bristol also recognised the disease independently in the same year. By 1930 it was also clear that ulcerative colitis was an utterly different disease from the dysenteries and that a bacterial cause was most unlikely.

In 1932 there was no understanding of the true nature of infective hepatitis. It was called catarrhal jaundice and was thought to be due to catarrh of the bile passages or an ascending infection from the duodenum. A general practitioner, Pickles, working in the Yorkshire Dales, was the first to show that hepatitis was an infectious, and sometimes an epidemic condition, with an exceptionally long incubation period of thirty days. The length of the incubation period and the occurrence of unrecognised anicteric cases had served to conceal the infectious character of the disease, but Pickles, by keeping meticulous records of the date of onset and location of every case was able to show the truth. It is a brilliant story of systematic observation and deduction, and one wonders what were the feelings of the few liver specialists in Europe who at that time were treating these cases by duodenal lavage with magnesium sulphate. The second world war coming soon afterwards forcibly drove home the epidemiological truth about this disease. In North Africa in 1941-42 an epidemic nearly brought both armies to a halt. It remained only for Sheila Sherlock and Professor Dible by utilizing the new technique of liver biopsy to show that the histological changes of this disease were widespread in every lobule of the liver and were maximal in the centrilobular area — well away from the bile ducts.

Returning to the inter-war years, we find surgeons tending to give up their misguided attempts to stitch up fallen organs, and some clear-minded debunking by a few convinced the many that an ‘ optosis ’ didn't necessarily call for an ‘ opexy ’. On the other hand, surgery for peptic ulcer was scarcely yet respectable. Gastroenterostomy, though often surprisingly successful, led too often to really ghastly complications such as gastrojejunocolic fistula, and gastrectomy pioneered by the Vienna School — Billroth and Polya — still seemed to many in those pre-antibiotic, rag-and-bottle-anaesthesia days to carry too high a mortality to be generally acceptable.

The physicians meanwhile continued to murder their cases of hæmatemesis by starving them, giving them ice to suck and letting them get dehydrated and uræmic. From Denmark the voice of Meulengracht, though seemingly iconoclastic, was listened to with respect and his greatly improved results obtained by liberal feeding of the hæmatemesis case gradually convinced even the most conservative. However, in those days, to give a blood transfusion was quite a labour, involving the recruitment and bleeding of willing relatives, while to give enough blood in the more severe cases was impossible. It was the coming of blood banks which revolutionised the management of hæmatemesis, and thus drastically reduced the mortality.

In 1937, forty-two years after a similar event in the U.S.A., an important moment arrived in the history of gastroenterology in Great Britain. Certain physicians who had been prominent in the investigation and treatment of gastroenterological conditions and who were attempting to bring the light of reason and experiment into this confused field, met in London and decided to start a British Society of Gastroenterology at whose annual meetings scientific papers would be read and clinical experiences shared. Wisely, the Society decided to open its membership to both surgeons, radiologists, and pathologists — to anyone, in fact, who professed an interest in the workings of the gastroenterological tract and who was willing to share his knowledge with his colleagues. The first president of this Society was the man who had been for years the intellectual leader of a team of physicians many of them from Guy's Hospital who had developed a critical and experimental approach to gastroenterological problems, Sir Arthur Hurst. Hurst was a man who exercised great influence over the minds of his contemporaries. He had a broad clinical grasp and a clear mind wishing to bring order out of chaos. This meant that sometimes he was wrong in his conclusions, which were derived only from accumulated experience of case histories; but his careful experiments and the fruitful observations of his radiological collaborator, Barclay, threw a great deal of light on many puzzling diseases. He showed that achalasia (he invented the name) was due to non-propulsion by the body of the œsophagus rather than to spasm of the sphincter at the lower end. He correctly deduced the presence of a physiological sphincter which was normally opened by the arrival of a peristaltic wave. In the absence of the peristaltic wave the sphincter wouldn't open. It is also interesting in these days to look back to his first description of the œsophagitis and stricture which might develop in the œsophagus above a hiatus hernia. His conclusion that the hernia developed because the œsophagus was short was, of course, wrong, but in his paper in the Quarterly Journal of Medicine of 1942 all the essential features of this disease can be found beautifully described. One sometimes hears it stated that the clinical syndromes associated with hiatus hernia would not have been elucidated without the interest, physiological, technical and pecuniary, of an enthusiastic band of thoracic surgeons. While recognizing the considerable new light which was thrown on this condition by surgical experimentation, it is also just to salute Sir Arthur Hurst for his early writings on the subject and it is realistic to give some credit to the radiologists who in the mid-forties were providing themselves with tilting X-ray tables which enabled them to demonstrate the lesion by barium. Much has been learned since then, but perhaps not yet the basic clinical rule that the presence of symptoms and an anatomical abnormality in the same patient does not necessarily mean that the former are due to the latter. In the practice of gastroenterology, it is wise to recognise that symptoms in the region of the upper gastro-intestinal tract may, in fact, arise from disease in the lower gastro-intestinal tract, or more important still from disease or disorder in the nervous system. In no branch of clinical science is it more important to assess the whole man, and the functions of the alimentary tract as a whole.

### SOME DEVELOPMENTS IN THE LAST 20 YEARS

We now come to the last twenty years in which there has been such a rapid growth in the knowledge of the gastro-intestinal tract, such an increase in enthusiasm for research, and such a large recruitment of young scientists to fields of gastroenterological study. To find a place for the publication of the results of excellent research work was becoming such a problem that in 1960 " Gut " the journal of the British Society of Gastroenterology was first published.

In these last twenty years gastric surgery has been established on a scientific basis, the aim of which in the treatment of duodenal ulcer is to reduce the potential acid secretory ability to a point at which recurrent ulceration is unlikely. In earlier days this meant cutting down the stomach to about a third of its normal size. However, the disadvantages of removing the " hopper junction " of the stomach, and of sending the food by a short cut into the jejunum so that it was not mixed properly with the pancreatic and biliary secretions, were soon appreciated by surgeons who ran proper follow-up clinics as was done here in Bristol. A certain influential medical knight once suggested that the surgeons in Bristol were showing an improper and exaggerated interest in the problems of the post-gastrectomy patients. But the careful studies of Mr. Butler and Mr. Capper at least analysed and defined the problem even if they didn't solve it. In 1954 Professor Kay of Glasgow utilized the fact that the newly discovered anti-histamine drugs did not inhibit that action of histamine which stimulates gastric acid secretion, to devise a test in which all the parietal cells of the stomach could be brought into activity at the same time. Thus, by giving a very large dose of histamine, while protecting the patient against its unpleasant side-effects by the prior administration of an anti-histamine drug, he was able to call forth a maximal secretory effort by the parietal cells. It has subsequently been shown by the painstaking studies of the Edinburgh gastroenterological team that this secretory effort is directly proportional to the number of parietal cells in the stomach. One can, therefore, possess a stomach with a large parietal cell population (2 billion), or one with a very small population. Those with the large parietal population may thus need more radical surgery than those with less.

The use of glass electrode pH monitoring showed in the early fifties that conventional antacids only raised the pH of the gastric juice for about ten minutes after they had been swallowed. At the same time follow-up studies showed that the long-term results of conventional medical treatment for duodenal ulcer were no better than " vis medicatrix naturæ ". What was needed, therefore, was some means of putting the parietal cell mass out of action, since the hyper-secretion of acid-pepsin mixture had long been proved to be the most important causative factor in duodenal ulcer. How to do this? Pharmacological blockage of the vagus nerve (known to be responsible for the psychic phase of gastric secretion) was precluded by the side-effects of adequate doses of atropine and other similar drugs. Vagotomy alone caused dangerous gastric stasis, but vagotomy and gastroenterostomy soon became a respectable operation.

### GASTRIN

However, the importance of the gastric antrum in the maintenance of the stimulus to gastric secretion was already realised. The gastric antrum is the most distal part of the stomach and does not itself secrete acid. In response to distension it produces a hormone, gastrin, which circulates through the blood and reaching by this route the fundus of the stomach stimulates the parietal cells. The presence of this hormone, gastrin, had long been suspected, but it was not until 1961 that Professor Gregory of Liverpool was able to obtain a reliable extract from the pig's antrum. Methods for assay of gastrin were also elaborated. Gastrin could also be produced by certain tumours of the pancreatic islets; and thus was explained a rare disease, the Zollinger Ellison syndrome, in which the stomach can produce over-night two litres or more of highly acid fluid which disturbs the functions of the small intestine and nearly always leads to multiple duodenal and jejunal ulceration. Gastrin is the most powerful known stimulus to parietal cell activity. The climax to this story comes in 1964 when Professor Gregory of Liverpool elucidated the chemical structure of gastrin. It is a polypeptide chain of 17 amino-acids. The astounding fact is that further studies in 1965 have shown that the pharmacological activity of the molecule is located in the last four amino-acids and the NH group. The gastrin story is a thrilling one in which the long-suspected hormone was first extracted and then in a very short time analysed. One can feel some pride that this brilliant and important work was done in Britain. Recently I.C.I. have synthesised a gastrin analogue containing the last four essential amino-acids of the gastrin molecule. This compound acts like gastrin but less powerfully. It is to be hoped that the chemists will before long provide us with an 'anti-gastrin' and then at last the medical treatment of duodenal ulcer will be on a sound basis.

### AETIOLOGY OF GASTRIC ULCER

Some very interesting studies have been going on in Bristol during the last five years which throw some light on the cause of that other form of peptic ulceration, gastric ulcer. Nowadays, gastric ulcer is much less common than duodenal ulcer, and the cause is likely to be different for the following reasons. The patient with duodenal ulcer often gives a family history of the same complaint, he has a 60% chance of being of blood group 0, and he has, as a rule, a large parietal cell population and a high maximal acid secretion. The patient with gastric ulcer has none of these features. He (or she) is often elderly, the stomach is usually affected by gastritis, and acid secretion is average or low. When operating on patients with duodenal ulcer and gastric ulcer Capper and Butler started to open the stomach and place strips of indicator paper over the mucosa so as to show which parts of the stomach were acid-secreting and which were alkaline-secreting (antrum). They examined a good number of cases and found a striking difference between the gastric ulcers and the duodenal ulcers. The duodenal ulcer patients had small antra and the gastric ulcer patients had large antra, but when the ulcer healed the parietal cell carpet might regrow over the scarred area. Furthermore, if the stomach was operated upon and a gastro-jejunal stoma fashioned, there was also an alkaline secreting zone around the stoma. This also happened with Billroth I, but not with Roux-Y anastamoses. The most likely explanation of this was that the regurgitation of biliary and pancreatic secretions in some way altered the characteristics of the gastric mucosa, so that parietal cells died out and were replaced by the antral type mucosa. Histological studies showed that this mucosa resembled antral mucosa, but had some evidence of chronic gastritis as well. Assays done by Chris Hallet in the Physiology Department showed that this antral type mucosa did not contain gastrin. Mr. Capper and Dr. Airth then devised a test designed to show whether, in fact, duodenal regurgitation occurred in the normal stomach. They placed a narrow tube in the duodenum which they filled with barium. They found that in most people the duodenal bulb or cap contracts very vigorously every minute or so, but when it does, the pylorus clamps down tight to prevent regurgitation. This closure of the pylorus occurs also in the uncomplicated case of duodenal ulcer, but most patients with active gastric ulcer show considerable regurgitation of barium into the stomach. Now duodenal juice containing trypsin, a proteolytic enzyme, is more irritant than gastric juice which depends for its proteolytic activity on a pH of 2 or less. It therefore seems reasonable to suggest that the gastritic changes found in this pseudo-antral type of mucosa might be caused by the irritant effect or regurgitated duodenal juice. Since gastritis appears to be an important cause of gastric ulceration, and since gastric ulcers tend to occur almost exclusively in the non-acid secreting zones of the stomach, it seems possible that duodenal regurgitation is an important factor either in the causation or in the perpetuation of gastric ulcer. At this stage it would be unwise to say more than this, but the working hypothesis seems an intriguing one which opens the way to further experiments to prove or disprove its truth.

### SMALL INTESTINAL MUCOSA IN THE COELIAC SYNDROME

Turning now to a different part of the alimentary canal—the small intestine—I would like to review some of the entirely new knowledge which has been gained in the last ten years. The story really begins in Holland during the war when Dicke in occupied Holland discovered that a wheat-free diet benefited children with coeliac disease. It was not long before three more things were discovered—that it was the gluten not the starch in wheat which caused the trouble, that the disease was much commoner in adults than had previously been suspected, and that adults too, if the disease was not too far advanced, would respond to a gluten-free diet. The next turning point was in 1956 when Dr. Margot Shiner working in Dr. Avery Jones's Department at the Central Middlesex Hospital found a reliable way of obtaining biopsies of jejunal mucosa, and showed conclusively that there was a distinct histological abnormality in patients with cœliac disease, idiopathic steatorrhoea, and sprue. The story of how she came to do this is worth retelling. Before the war Thaysen had written a very good paper on the clinical features of steatorrhoea. In it he stated that there was no proven histological lesion in this disease. He based this statement on *post mortem* histology which in the stomach and small intestine is utterly unreliable because as soon as death occurs the digestive ferments present within the lumen of the gut begin to digest away the mucosa. However, because his paper was well written and authoritative, it was quoted widely and the erroneous idea found its way into every textbook. About 1951 Dr. J. W. Paulley, a physician of the Regional Board Hospital in Ipswich, previously a registrar of Dr. Avery Jones, began to turn up at the meetings of the British Society of Gastroenterology and the Medical Research Society with microscopic slides in his pocket which he invited those whom he could interest to gaze upon. These slides were of biopsy specimens obtained from the jejunum at the time of laparotomy. One was from a patient with idiopathic steatorrhœa and one was from a patient with appendicitis. As one who was asked to gaze down a microscope at these slides I can report that the contrast between the two specimens was remarkable. The one showed long, fine villi, the other showed no proper villi, but only the blunted stumps buried in a thick submucosa. Others also were convinced, including Dr. Avery Jones, who put the idea to Dr. Shiner that she should try to develop a modification of the biopsy knife developed by the late Ian Wood of Melbourne for taking specimens of gastric mucosa. By utilizing Bowden cable such as is used for the 'choke' mechanism of the carburettor or clutch of a motor cycle, she was able to operate the biopsy knife at the end of a three foot tube which was (just) deglutible. The mucosa was sucked into a round hole in the apparatus by a syringe at the mouth end; then the knife cut off a small circular piece. The same principle is used by subsequent biopsy tools, but the knife can now be operated by pneumatic or hydraulic means, thus making the tube much smaller and more easily deglutible.

The histological lesion of tropical sprue, steatorrhœa, and cœliac disease is the same. Although the treatment of tropical sprue is based on the use of antibiotics and removal to a temperate climate, it can be stated as a broad generalisation that the presence of a flat mucosal biopsy makes it likely that a gluten-free diet will cure the patient's diarrhœa, decrease the degree of malabsorption, and improve his nutritional status. The absence of a flat mucosal biopsy in any patient with proven steatorrhœa and malabsorption makes it probable that the malabsorption is secondary to some other disease : a stricture of the gut, an entero-colic fistula, a blind loop, pancreatitis—and many others. In this way, jejunal biopsy is an important diagnostic procedure.

About 1961 Dr. Alan Read asked me if a certain patient of mine suffering from idiopathic steatorrhœa had had a 'flat mucosa' when the biopsy was examined two years previously. The reason for his question was that the patient had developed a reticulo-sarcoma of his small intestine. This was the beginning of an interesting piece of investigation done by Dr. Read and Dr. Gough during the succeeding three years. First, they found seven cases in Bristol in whom the diagnosis of idiopathic steatorrhœa had been soundly based on jejunal biopsy or length of history stretching back into childhood. Then by searching the world literature and by making enquiries at centres throughout this country, they were able to 'dig up' a total of twenty-three cases in which reticulo-sarcoma, Hodgkin's disease, or lymphosarcoma appeared to develop in a patient previously known to be suffering from idiopathic steatorrhœa. Previously, doctors who had noticed the association had concluded that the steatorrhœa was due to the reticulo-sarcoma invading the gut or interfering with its absorptive channels. The evidence now brought forward by Gough and Read disposed of this possibility, and so we have evidence of a disease predisposing to a reticulosis. We know of many gastro-intestinal diseases—gastritis, polyposis, ulcerative coltis—which predispose to carcinoma, but here is a mucosal abnormality which may lead to a tumour of reticulo-endothelial origin. It is interesting to speculate as to how this could happen.

The dynamics of intestinal mucosal growth and replacement are fascinating. The researches of Creamer of St. Thomas's Hospital and others have shown that the villi (of which there must be several millions) are in a continual state of growth and destruction. The germ cell lies deep in the crypts : the daughter cells budded off form a queue which moves methodically outwards towards the tip of the villus. Such a cell lives at the most three days and is then shed into the lumen of the gut. Presumably its active life of absorption may last only a day, but what a chemical engine this cell is! It has been shown recently to contain enzymes for the splitting of sugars, for the uptake of many substances against an osmotic gradient, and for the mysterious process of fat absorption and transport.

In the sprue syndrome what happens? The rate of cell turnover is decreased, and the relatively few so formed have to work much harder and to go on working when they are quite worn out. With such a state of disorganization in cells of such dynamism, it is easy to imagine that cancer could develop. It is not cancer that develops, but a disease of the reticulum, the connective tissue, the mesoderm. There is a possible clue as to how this could happen, and that is the finding by Dr. McCarthy of Bristol that in some of these patients there is a notable shrinkage of lymphatic tissue; spleen, lymph nodes and lymphoid follicles are in some cases very shrunken. He has shown that in these patients—a minority of those with idiopathic steatorrhœa—there is not only shrinkage of the reticulo-endothelial system, but a failure of some of its functions.

It is clear that in this field as in every other, what we don't know far exceeds what we do know, and that every question answered delineates many more yet to be answered. It will, I think, be generally agreed that the rate of growth in gastroenterology is at least as fast as in most other fields of medical enquiry and I can assure you that here in Bristol the growing points are being carefully tended.

